# Radiation-Induced Left Main Coronary Artery Stenosis in a Patient with Atretic Internal Mammary Arteries

**DOI:** 10.1155/2020/7970305

**Published:** 2020-03-11

**Authors:** Siddhant Dogra, Asha M. Mahajan, Albert Jung, Michael Attubato, Muhamed Saric, Alan Shah

**Affiliations:** Leon H. Charney Division of Cardiology, Department of Medicine, New York University School of Medicine, 530 First Avenue, Skirball 9U, New York, NY10016, USA

## Abstract

Coronary artery disease (CAD) is a known potential complication of thoracic radiation treatment that typically affects the proximal segments of the coronary arteries, requiring coronary artery bypass grafting (CABG). We present a case of acute coronary syndrome occurring in a 57-year-old man with prior thoracic radiation therapy following resection of a chest wall chondrosarcoma. Coronary angiogram demonstrated significant areas of stenosis in the left main coronary artery (LMCA) and ostial left anterior descending (LAD) coronary artery. The patient was also found to have atretic bilateral internal mammary arteries as a consequence of his radiation therapy, rendering them unsuitable as grafts. Percutaneous coronary intervention (PCI) was thus performed with a successful outcome. To our knowledge, this is the first case of radiation-induced CAD of the LMCA with atretic internal mammary arteries treated successfully with PCI.

## 1. Introduction

Radiation therapy can play a critical role in treatment of cancers involving the chest such as breast cancer, lung cancer, lymphoma, and other thoracic-related cancers. However, radiation therapy does pose a risk of damaging pulmonary and cardiovascular tissues. Radiation-induced cardiovascular disease can manifest as acute or constrictive pericarditis, aortic disease, valvular disease, heart failure, arrhythmias, and vasculopathy involving both microvascular and macrovascular structures, including coronary artery disease (CAD). The injury can either be acute or develop years after treatment [1]. Studies in patients receiving radiation therapy suggest a dose-response relationship between radiation exposure and incidence of cardiovascular complications [2]. Radiation-induced CAD tends to affect the left main coronary artery (LMCA) or proximal left anterior descending (LAD) artery, making a patient more favorable for coronary artery bypass grafting (CABG) [3]. However, radiation may also damage the internal mammary arteries. In this report, we describe to our knowledge the first case of a patient with radiation-induced LMCA disease with atretic left and right internal mammary arteries treated successfully with percutaneous coronary intervention (PCI).

## 2. History of Presentation and Medical History

**Figure 1:**
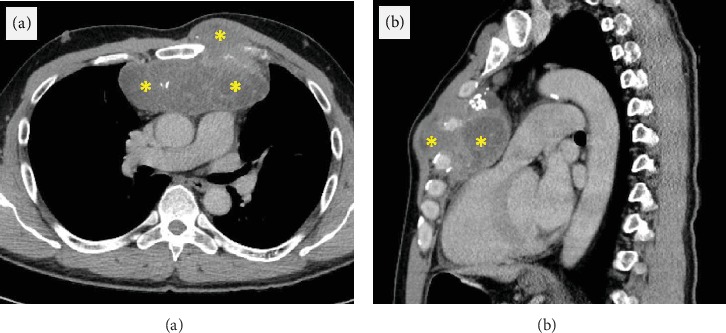
Chest wall chondrosarcoma on prior CT scan. CT scan of chest wall chondrosarcoma in (a) transverse and (b) sagittal planes marked by asterisks.

A 57-year-old man presented to his outpatient cardiologist with two weeks of exertional left-sided chest pain and decrease in exercise tolerance from a baseline of 20 blocks to 1 block. The pain was described as pressure in a band-like region across his chest, brought on by exertion and relieved after 5-10 minutes of rest. His history was significant for high-grade dedifferentiated chest wall chondrosarcoma involving the left ribs that was resected three years earlier (Figures 1(a) and 1(b)). The internal mammary arteries were isolated and protected perioperatively. The patient subsequently received one cycle of adjuvant high-dose ifosfamide with mesna complicated by worsening renal function. This was followed by thoracic radiation treatment of 60 Gray total delivered in 30 fractions. He then was started on pazopanib daily complicated by hypertension and hypothyroidism.

## 3. Investigation

The patient experienced chest pain walking to the exam room and was sent to the emergency department for further evaluation. His electrocardiogram showed normal sinus rhythm with new T-wave inversions in the anterior and lateral leads as well as poor R wave progression. Two successive troponins were elevated at 0.13 and 0.11 ng/mL. A transthoracic echocardiogram showed severe hypokinesis in the LAD distribution with a new mildly reduced ejection fraction of 40%.

**Figure 2:**
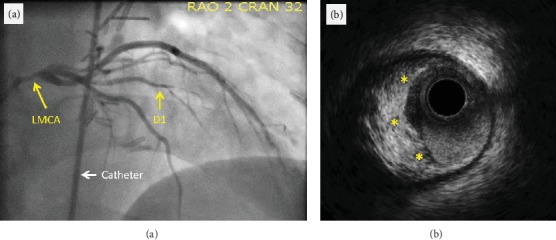
Initial coronary angiogram of left coronary system. Coronary angiogram showing the stenotic lesion in the (a) left internal mammary artery (LMCA) and first diagonal branch (D1) and (b) IVUS of LMCA lesion showing significant plaque (noted by yellow asterisks).

**Figure 3:**
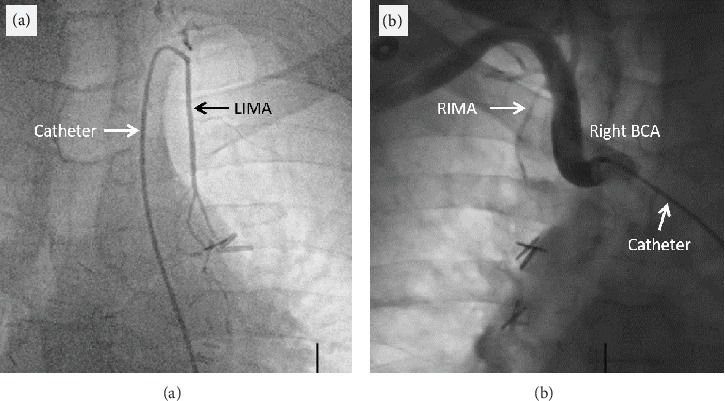
Coronary angiogram of internal mammary arteries. Coronary angiogram of the (a) left internal mammary artery (LIMA) and the (b) right internal mammary artery (RIMA) coming just off of the right brachiocephalic artery (BCA).

Coronary angiogram showed significant LMCA stenosis extending into the ostial segment of the LAD artery with 90% stenosis, which was further evaluated by intravascular ultrasound (IVUS) noting significant plaque (Figures 2(a) and 2(b)). There were also stenotic lesions in the first diagonal branch. The right coronary artery, left circumflex artery, and ramus intermedius artery displayed minimal luminal abnormalities. Given the patient's history of chest radiation therapy, angiography of the internal mammary arteries was performed and demonstrated diminutive, possibly occluded vessels (Figures 3(a) and 3(b)).

## 4. Management

**Figure 4:**
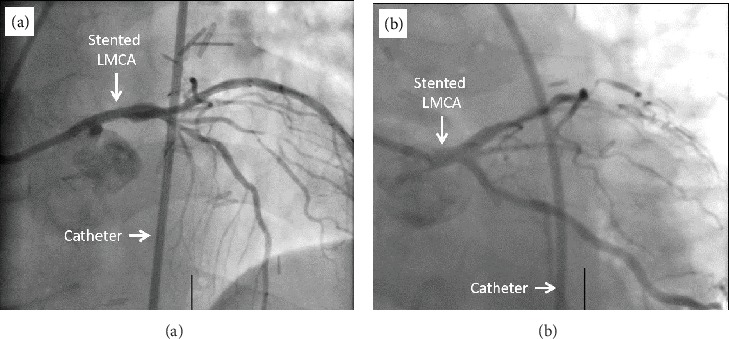
Coronary angiogram of LMCA lesion after PCI. (a, b) Coronary angiogram of LMCA lesion after stent placement.

PCI was performed on the mid-LMCA to ostial LAD lesion. After prestent dilatation, a 3.5 × 20 mm Promus ELITE drug-eluting stent was successfully delivered (Figures 4(a) and 4(b)). Poststent balloon dilatation was performed, and IVUS confirmed good stent apposition and expansion. The decision was made to medically manage the first diagonal branch disease. The patient was discharged on aspirin 81 mg daily, ticagrelor 90 mg twice daily, and atorvastatin 80 mg daily in addition to continuing his home medications.

## 5. Follow-Up

Three months postdischarge, the patient was doing well, asymptomatic, and back to his baseline exercise tolerance.

## 6. Discussion

Cardiovascular disease and cancer together are the top two leading causes of mortality worldwide and share significant common characteristics including overlap in risk factors as well as pathophysiologic components ranging from genetic to inflammatory. Moreover, numerous cancer treatments have been demonstrated to be cardiotoxic, including chemotherapeutic agents such as doxorubicin and newer targeted immunotherapies. Combination with radiation can increase the risk of cardiac toxicities. As cancer therapies have improved, cancer survival rates have significantly increased. As the number of cancer survivors grows, practitioners must be aware of the links between cancer treatment and development of future cardiac complications [4].

Radiation therapy can affect both pulmonary and cardiovascular structures and function. Patients can develop pulmonary toxicities such as radiation-induced fibrosis or pleural effusions, which can be both acute and chronic. Pulmonary hypertension can develop as a consequence of this injury. They can develop vasculopathy which can involve both macrovascular and microvascular structures. There is a risk of developing aortic calcification which can complicate surgical options. CAD and microvascular disease can lead to both angina and ischemic cardiomyopathy. Patients can develop both acute and chronic pericarditis, pericardial effusions, and constrictive pericarditis. Valvular heart disease usually presents as progressive thickening and calcification which can affect the valvular structure, the subvalvular apparatus, and the aortomitral curtain. The aortic and mitral valves are more commonly affected, and symptomatic disease can develop 10 to 20 years later [5]. Conduction defects including atrial fibrillation, atrioventricular block, and ventricular arrhythmias can also occur. Finally, radiation can cause myocardial dysfunction due to diffuse fibrosis and can lead to heart failure [6, 7].

Thoracic radiation treatment can lead to accelerated CAD, even in patients without typical risk factors. Patients with radiation-induced CAD tend to present at a younger age than the general population. They commonly have LMCA and/or proximal LAD lesions since these structures are more anterior and central and thus likely exposed to more radiation than distal vessel segments [1, 3]. Radiation-induced CAD is thought to be driven by increased inflammatory cytokine production and inflammatory cell recruitment leading to accelerated intimal plaque formation. Furthermore, radiation induces fibrosis of the media and adventitia in the vessel wall. Inflammatory marker concentration and risk of vessel injury increase proportionally to cumulative radiation dose [1].

Radiation-induced CAD management in terms of medical therapy and revascularization remains the same as that of atherosclerotic CAD. However, revascularization decisions can become challenging as radiation can damage the internal mammary arteries that are more favorable to use especially for left main and LAD disease [1, 8]. Some concern exists about whether the internal mammary arteries should be used as grafts even if they appear undamaged due to potential radiation-induced atherosclerosis. It has been reported that the use of healthy internal mammary arteries is not associated with early graft failure up to one year [9]. However, there was a reported case of a patient with radiation-induced CAD who underwent CABG using the internal mammary arteries and decompensated immediately postoperatively. Emergent angiography showed suboccluded arterial grafts, which were explanted and replaced by saphenous vein grafts. Pathological analysis of the arterial grafts showed significant atherosclerotic plaque and fibrosis [10]. In addition, among patients with radiation-induced CAD who undergo surgery, there is higher risk of postoperative complications such as poor wound healing and higher mortality up to 10 years compared to CABG patients with no history of radiation therapy [11]. PCI is an appealing alternative as it has been shown to be noninferior to CABG for LMCA disease among patients with low-intermediate SYNTAX scores [12]. However, mortality is reportedly higher in patients who undergo PCI for radiation-induced CAD compared to those without radiation exposure at up to 12 years, and there is debate about whether restenosis rates may be higher [13].

Given our patient's significant LMCA stenosis with bifurcation involvement to the LAD and low disease burden in other vessels, it would be most appropriate to pursue CABG based on the 2017 ACC/AHA Appropriate Use Criteria [14]. However, our patient had atretic left and right internal mammary arteries due to radiation, making them unfavorable grafts. In addition, he would have had increased risk of sternal wound infection given this would have been his second sternotomy in the setting of prior radiation therapy. Ultimately, our patient underwent PCI to the lesion with an excellent angiographic result.

## 7. Conclusion

CAD is one possible consequence of thoracic radiation treatment. The cardiovascular team should evaluate both pulmonary and cardiovascular complications of radiation in order to determine the best revascularization strategy that will lead to a good long-term patency as well as reduce comorbidities of the revascularization procedure. While CABG is commonly used, patients may have radiation damage to the internal mammary arteries. PCI is an available option in the treatment of these patients.

## Abbreviations

CAD: Coronary artery disease
CABG: Coronary artery bypass grafting
LMCA: Left main coronary artery
PCI: Percutaneous coronary intervention
LAD: Left anterior descending
IVUS: Intravascular ultrasound
D1: First diagonal branch
LIMA: Left internal mammary artery
RIMA: Right internal mammary artery
BCA: Brachiocephalic artery
